# Supplementary material for: Geochemical characteristics of dissolved heavy metals in Zhujiang River, Southwest China: spatial-temporal distribution, source, export flux estimation, and a water quality assessment
Source: PeerJ. 2019 Mar 13;7:e6578. doi: 10.7717/peerj.6578 (PMC6420802; doi:10.7717/peerj.6578)
Supplement: Supplemental Information 1 — NPR, Nanpanjiang River; HSR, Hongshuihe River; QJR, Qianjiang River; XUJ, Xunjiang River; XJR, Xijiang River.T-NPR, T-BPR, T-HSR, T-QJR, T-XUJ and T-XJR represents the tributaries of Nanpanjiang River, Beipanjiang River, Hongshuihe River, Qianjiang River, Xunjiang River and Xijiang River. [file peerj-07-6578-s001.docx]

| Site number | River reach | Sample location | Date (m-d-y) | WQI | V | Cr | Mn | Co | Ni | Cu | Mo | Cd | Ba | Pb |
| --- | --- | --- | --- | --- | --- | --- | --- | --- | --- | --- | --- | --- | --- | --- |
|  |  |  |  |  | μg L^-1^ | μg L^-1^ | μg L^-1^ | μg L^-1^ | μg L^-1^ | μg L^-1^ | μg L^-1^ | μg L^-1^ | μg L^-1^ | μg L^-1^ |
| High flow season | | | | | | | | | | | | | | |
| 1 | NPR | N：25º54.376' E：103º57.101' | 07/18/14 | 8.3 | 2.11 | 4.75 | 11.36 | 0.08 | 10.03 | 68.86 | 0.13 | 0.03 | 4.00 | 0.07 |
| 2 | NPR | N：25º36.971' E：103º49.532' | 07/19/14 | 7.6 | 2.57 | 10.48 | 0.39 | 0.16 | 8.50 | 50.30 | 0.40 | 0.02 | 14.78 | 0.08 |
| 3 | NPR | N：25º17.512' E：103º51.405' | 07/19/14 | 6.7 | 2.81 | 11.66 | 0.76 | 0.14 | 6.81 | 4.30 | 0.40 | 0.03 | 26.09 | 0.04 |
| 4 | NPR | N：25º00.589' E：103º38.313' | 07/19/14 | 5.1 | 2.97 | 13.90 | 0.40 | 0.15 | 2.75 | 1.04 | 0.55 | 0.03 | 19.72 | 0.06 |
| 5 | NPR | N：24º56.169' E：103º10.674' | 07/19/14 | 4.1 | 3.09 | 8.30 | 1.62 | 0.13 | 2.80 | 0.96 | 0.78 | 0.03 | 28.39 | 0.03 |
| 6 | NPR | N：24º13.314' E：103º06.415' | 07/20/14 | 4.8 | 3.07 | 11.88 | 0.16 | 0.19 | 2.84 | 1.11 | 0.85 | 0.03 | 24.13 | 0.05 |
| 7 | T-NPR | N：24º12.664' E：103º06.806' | 07/20/14 | 11.7 | 3.34 | 9.97 | 0.22 | 0.17 | 16.72 | 20.43 | 0.78 | 0.05 | 48.40 | 0.08 |
| 8 | T-NPR | N：24º13.778' E：103º23.804' | 07/19/14 | 27.9 | 3.48 | 14.87 | 0.47 | 0.19 | 49.03 | 29.02 | 0.52 | 0.03 | 20.56 | 0.05 |
| 9 | T-NPR | N：24º39.257' E：104º28.790' | 07/20/14 | 4.0 | 2.43 | 10.85 | 0.14 | 0.11 | 2.38 | 1.17 | 0.41 | 0.03 | 8.22 | 0.04 |
| 10 | NPR | N：24º39.293' E：104º28.732' | 07/20/14 | 4.1 | 2.63 | 10.85 | 0.14 | 0.14 | 2.31 | 1.81 | 0.93 | 0.03 | 13.94 | 0.04 |
| 11 | T-NPR | N：24º46.524' E：104º32.253' | 07/20/14 | 6.3 | 2.56 | 9.54 | 0.13 | 0.11 | 7.68 | 5.29 | 0.43 | 0.04 | 10.73 | 0.04 |
| 12 | T-NPR | N：25º11.607' E：104º54.663' | 07/21/14 | 3.8 | 2.66 | 6.40 | 0.67 | 0.14 | 3.85 | 0.87 | 0.50 | 0.04 | 9.14 | 0.07 |
| 13 | NPR | N：24º52.129' E：105º01.755' | 07/21/14 | 5.0 | 2.81 | 7.69 | 0.16 | 0.08 | 5.80 | 1.22 | 0.90 | 0.03 | 12.33 | 0.03 |
| 14 | T-BPR | N：26º00.141' E：104º30.067' | 07/18/14 | 14.7 | 2.50 | 8.48 | 0.36 | 0.09 | 24.73 | 5.43 | 0.81 | 0.04 | 33.90 | 0.04 |
| 15 | T-BPR | N：26º17.388' E：104º42.789' | 07/18/14 | 21.3 | 2.86 | 6.89 | 0.57 | 0.09 | 37.46 | 95.66 | 0.62 | 0.03 | 13.39 | 0.09 |
| 16 | T-BPR | N：26º22.931' E：105º00.733' | 07/17/14 | 18.2 | 2.36 | 5.19 | 134.49 | 0.51 | 2.03 | 0.53 | 0.96 | 0.04 | 13.49 | 0.04 |
| 17 | T-BPR | N：26º26.503' E：105º04.567' | 07/17/14 | 3.2 | 2.52 | 7.67 | 0.53 | 0.12 | 1.95 | 0.37 | 0.58 | 0.06 | 13.46 | 0.03 |
| 18 | T-BPR | N：26º10.138' E：105º11.266' | 07/17/14 | 2.3 | 2.78 | 5.75 | 0.23 | 0.09 | 1.22 | 0.46 | 0.76 | 0.03 | 13.00 | 0.03 |
| 19 | T-BPR | N：25º40.338' E：105º39.591' | 07/17/14 | 3.6 | 2.28 | 8.94 | 0.41 | 0.12 | 2.09 | 0.45 | 0.60 | 0.02 | 11.30 | 0.12 |
| 20 | T-BPR | N：26º00.774' E：105º40.083' | 07/17/14 | 10.3 | 2.40 | 9.13 | 0.78 | 0.13 | 12.03 | 136.80 | 0.52 | 0.03 | 24.75 | 0.09 |
| 21 | T-BPR | N：25º53.339' E：105º38.595' | 07/17/14 | 14.6 | 2.13 | 7.97 | 49.67 | 0.20 | 11.21 | 103.17 | 0.94 | 0.03 | 9.14 | 0.04 |
| 22 | T-BPR | N：25º52.383' E：105º40.367' | 07/17/14 | 3.5 | 2.34 | 8.90 | 0.76 | 0.12 | 1.89 | 0.53 | 0.63 | 0.03 | 17.78 | 0.04 |
| 23 | T-BPR | N：25º31.932' E：105º45.989' | 07/16/14 | 3.2 | 2.30 | 7.66 | 0.22 | 0.12 | 2.02 | 0.58 | 0.60 | 0.03 | 15.03 | 0.04 |
| 24 | T-BPR | N：25º28.012' E：105º46.908' | 07/16/14 | 3.4 | 2.37 | 8.17 | 1.32 | 0.14 | 1.98 | 1.81 | 0.66 | 0.03 | 13.80 | 0.04 |
| 25 | T-BPR | N：25º23.054' E：105º45.477' | 07/16/14 | 3.6 | 2.53 | 9.94 | 0.26 | 0.14 | 1.84 | 0.51 | 0.53 | 0.03 | 14.97 | 0.04 |
| 26 | T-BPR | N：25º22.678' E：105º47.127' | 07/16/14 | 4.0 | 2.40 | 7.93 | 6.41 | 0.18 | 2.12 | 0.58 | 0.68 | 0.03 | 14.03 | 0.04 |
| 27 | T-BPR | N：25º06.965' E：105º51.618' | 07/15/14 | 5.1 | 2.10 | 14.96 | 0.60 | 0.26 | 2.15 | 0.24 | 0.20 | 0.03 | 20.51 | 0.07 |
| 28 | T-BPR | N：25º07.095' E：105º51.757' | 07/15/14 | 5.0 | 2.21 | 13.33 | 0.22 | 0.27 | 2.15 | 0.30 | 0.25 | 0.03 | 40.60 | 0.05 |
| 29 | T-BPR | N：25º04.399' E：105º57.121' | 07/15/14 | 3.6 | 2.33 | 8.50 | 0.17 | 0.14 | 2.20 | 0.47 | 0.68 | 0.03 | 19.88 | 0.04 |
| 30 | T-BPR | N：25º02.840' E：105º55.993' | 07/15/14 | 3.3 | 2.19 | 8.94 | 0.56 | 0.17 | 1.48 | 0.42 | 0.40 | 0.02 | 12.98 | 0.11 |
| 31 | T-BPR | N：24º09.598' E：106º05.519' | 07/15/14 | 7.0 | 1.96 | 12.55 | 22.84 | 0.36 | 1.88 | 0.41 | 0.42 | 0.03 | 17.87 | 0.07 |
| 32 | T-BPR | N：24º56.984' E：106º08.133' | 07/15/14 | 3.5 | 2.25 | 7.89 | 0.26 | 0.10 | 2.29 | 0.64 | 0.73 | 0.04 | 15.49 | 0.14 |
| 33 | NPR | N：24º56.894' E：106º08.749' | 07/15/14 | 3.6 | 2.14 | 7.56 | 0.26 | 0.10 | 2.33 | 0.52 | 0.63 | 0.08 | 11.93 | 0.22 |
| 34 | HSR | N：24º59.741' E：106º09.535' | 07/15/14 | 5.9 | 2.13 | 9.40 | 0.54 | 0.11 | 5.93 | 8.71 | 0.54 | 0.04 | 32.45 | 0.06 |
| 35 | T-HSR | N：24º58.122' E：106º09.510' | 07/15/14 | 10.7 | 2.39 | 7.45 | 0.31 | 0.10 | 16.79 | 27.44 | 0.77 | 0.03 | 22.60 | 0.06 |
| 36 | HSR | N：25º09.847' E：106º37.839' | 07/24/14 | 2.7 | 2.69 | 6.99 | 0.24 | 0.10 | 1.43 | 0.59 | 0.69 | 0.04 | 10.38 | 0.04 |
| 37 | T-HSR | N：26º05.865' E：106º38.245' | 07/24/14 | 5.2 | 2.50 | 10.39 | 8.11 | 0.23 | 3.07 | 0.45 | 0.96 | 0.03 | 9.08 | 0.04 |
| 38 | T-HSR | N：25º27.515' E：106º46.701' | 07/24/14 | 3.6 | 2.34 | 9.21 | 1.51 | 0.10 | 2.04 | 0.47 | 0.26 | 0.03 | 4.34 | 0.06 |
| 39 | T-HSR | N：25º04.082' E：106º57.780' | 07/24/14 | 2.9 | 2.40 | 5.10 | 0.18 | 0.08 | 2.82 | 6.76 | 0.35 | 0.03 | 5.62 | 0.03 |
| 40 | HSR | N：24º59.919' E：107º10.015' | 07/24/14 | 3.4 | 2.51 | 8.27 | 0.22 | 0.11 | 2.32 | 1.16 | 0.56 | 0.04 | 10.47 | 0.04 |
| 41 | T-HSR | N：24º50.647' E：107º19.850' | 07/25/14 | 3.8 | 2.34 | 5.83 | 0.71 | 0.10 | 3.15 | 13.52 | 0.35 | 0.20 | 8.34 | 0.06 |
| 42 | T-HSR | N：24º31.637' E：107º45.463' | 07/25/14 | 4.2 | 2.40 | 9.91 | 0.58 | 0.12 | 3.17 | 1.26 | 0.22 | 0.03 | 5.76 | 0.05 |
| 43 | T-HSR | N：24º34.169' E：107º49.052' | 07/25/14 | 12.0 | 2.34 | 6.10 | 47.07 | 0.19 | 3.41 | 1.02 | 0.59 | 1.36 | 8.92 | 0.22 |
| 44 | T-QJR | N：24º42.316' E：108º02.125' | 07/25/14 | 3.5 | 2.42 | 8.78 | 0.17 | 0.10 | 2.08 | 0.73 | 0.36 | 0.11 | 6.69 | 0.04 |
| 45 | T-QJR | N：24º34.145' E：108º28.336' | 07/25/14 | 3.5 | 2.40 | 8.84 | 0.34 | 0.11 | 2.12 | 0.52 | 0.30 | 0.07 | 7.69 | 0.03 |
| 46 | T-QJR | N：24º30.130' E：108º37.738' | 07/25/14 | 3.2 | 2.43 | 7.51 | 0.24 | 0.11 | 2.29 | 0.51 | 0.32 | 0.07 | 8.04 | 0.03 |
| 47 | T-QJR | N：24º32.325' E：109º14.632' | 07/26/14 | 3.3 | 2.54 | 8.18 | 0.13 | 0.10 | 2.05 | 0.45 | 0.31 | 0.07 | 7.92 | 0.04 |
| 48 | T-QJR | N：24º32.698' E：109º15.904' | 07/26/14 | 1.7 | 2.66 | 2.80 | 0.70 | 0.04 | 1.19 | 3.66 | 0.28 | 0.04 | 11.02 | 0.03 |
| 49 | T-QJR | N：24º32.082' E：109º14.962' | 07/26/14 | 2.8 | 2.46 | 5.95 | 0.29 | 0.06 | 2.30 | 0.50 | 0.29 | 0.05 | 7.97 | 0.03 |
| 50 | T-QJR | N：23º50.551' E：109º31.877' | 07/26/14 | 2.9 | 2.84 | 5.11 | 1.48 | 0.07 | 2.06 | 0.71 | 0.40 | 0.07 | 19.63 | 0.05 |
| 51 | QJR | N：23º40.025' E：109º40.139' | 07/26/14 | 6.5 | 2.39 | 7.37 | 0.33 | 0.10 | 8.45 | 27.32 | 0.35 | 0.07 | 10.14 | 0.04 |
| 52 | QJR | N：23º36.249' E：109º38.862' | 07/26/14 | 3.5 | 2.46 | 8.85 | 0.42 | 0.14 | 1.87 | 0.48 | 0.39 | 0.08 | 10.50 | 0.04 |
| 53 | QJR | N：23º24.696' E：110º04.310' | 07/27/14 | 3.8 | 2.46 | 7.52 | 0.11 | 0.08 | 3.25 | 0.77 | 0.35 | 0.07 | 12.37 | 0.04 |
| 54 | T-XUR | N：22º49.966' E：108º05.609' | 07/31/14 | 8.1 | 2.73 | 7.87 | 0.32 | 0.39 | 12.14 | 8.89 | 0.16 | 0.02 | 11.93 | 0.03 |
| 55 | T-XUR | N：22º50.682' E：108º05.743' | 07/31/14 | 3.1 | 3.53 | 8.26 | 0.18 | 0.11 | 1.84 | 0.74 | 0.62 | 0.02 | 10.11 | 0.02 |
| 56 | T-XUR | N：22º49.395' E：108º11.372' | 07/31/14 | 3.7 | 3.31 | 8.15 | 0.78 | 0.12 | 1.96 | 0.52 | 0.46 | 0.03 | 11.69 | 0.45 |
| 57 | T-XUR | N：22º54.480' E：109º34.174' | 07/30/14 | 2.9 | 3.18 | 6.62 | 0.19 | 0.10 | 1.99 | 0.84 | 0.43 | 0.04 | 13.46 | 0.05 |
| 58 | T-XUR | N：23º22.370' E：110º04.851' | 07/27/14 | 2.5 | 2.73 | 4.93 | 3.64 | 0.08 | 1.08 | 0.67 | 0.38 | 0.03 | 11.39 | 0.05 |
| 59 | XUR | N：23º26.103' E：110º07.647' | 07/27/14 | 2.5 | 2.75 | 6.61 | 0.19 | 0.12 | 1.23 | 0.56 | 0.31 | 0.03 | 11.16 | 0.03 |
| 60 | XUR | N：23º25.594' E：110º31.940' | 07/27/14 | 2.7 | 3.06 | 6.59 | 0.12 | 0.08 | 1.54 | 0.65 | 0.43 | 0.03 | 13.67 | 0.04 |
| 61 | T-XUR | N：23º24.980' E：110º31.817' | 07/27/14 | 3.8 | 2.64 | 5.18 | 16.58 | 0.08 | 0.60 | 0.48 | 0.15 | 0.03 | 12.50 | 0.08 |
| 62 | XUR | N：23º25.359' E：110º32.275' | 07/27/14 | 2.5 | 2.90 | 6.40 | 0.17 | 0.07 | 1.33 | 0.46 | 0.36 | 0.03 | 12.58 | 0.03 |
| 63 | XUR | N：23º21.571' E：110º51.468' | 07/27/14 | 2.5 | 3.08 | 6.26 | 0.24 | 0.09 | 1.40 | 0.66 | 0.44 | 0.03 | 14.29 | 0.04 |
| 64 | T-XUR | N：23º20.937' E：110º53.646' | 07/27/14 | 2.5 | 2.66 | 1.82 | 12.16 | 0.12 | 0.56 | 1.08 | 0.22 | 0.02 | 16.94 | 0.04 |
| 65 | XUR | N：23º23.557' E：110º55.022' | 07/27/14 | 3.0 | 2.86 | 7.00 | 1.29 | 0.08 | 1.68 | 1.30 | 0.40 | 0.03 | 13.50 | 0.05 |
| 66 | T-XJR | N：23º30.364' E：110º19.704' | 07/27/14 | 1.7 | 2.59 | 3.93 | 0.27 | 0.04 | 0.89 | 0.59 | 0.31 | 0.02 | 11.56 | 0.03 |
| 67 | XJR | N：23º28.637' E：110º20.612' | 07/27/14 | 2.7 | 3.00 | 6.52 | 0.94 | 0.08 | 1.37 | 0.55 | 0.44 | 0.02 | 14.13 | 0.04 |
| 68 | T-XJR | N：23º27.087' E：111º30.237' | 07/28/14 | 3.2 | 2.58 | 2.94 | 9.42 | 0.05 | 2.27 | 0.85 | 0.28 | 0.04 | 9.03 | 0.10 |
| 69 | XJR | N：23º20.329' E：111º31.631' | 07/28/14 | 2.4 | 3.02 | 5.55 | 0.73 | 0.09 | 1.35 | 0.71 | 0.42 | 0.04 | 12.95 | 0.05 |
| 70 | T-XJR | N：23º07.208' E：111º49.460' | 07/28/14 | 1.3 | 2.91 | 2.85 | 0.40 | 0.04 | 0.67 | 0.65 | 0.28 | 0.03 | 8.85 | 0.04 |
| 71 | T-XJR | N：23º09.157' E：111º51.424' | 07/28/14 | 2.3 | 2.76 | 2.32 | 8.88 | 0.05 | 0.64 | 1.48 | 0.27 | 0.02 | 13.47 | 0.08 |
| 72 | XJR | N：23º07.908' E：111º55.040' | 07/28/14 | 3.1 | 3.36 | 7.33 | 0.35 | 0.14 | 1.55 | 0.84 | 0.53 | 0.05 | 22.54 | 0.04 |
| 73 | T-XJR | N：23º05.709' E：112º07.908' | 07/28/14 | 3.3 | 2.77 | 1.98 | 19.35 | 0.04 | 0.47 | 0.62 | 0.22 | 0.03 | 11.05 | 0.06 |
| 74 | T-XJR | N：23º11.024' E：112º19.212' | 07/28/14 | 5.7 | 2.87 | 2.55 | 38.78 | 0.12 | 0.69 | 1.03 | 0.34 | 0.03 | 8.63 | 0.06 |
| 75 | XJR | N：23º02.455' E：112º25.236' | 07/29/14 | 2.8 | 3.23 | 7.04 | 0.35 | 0.15 | 1.49 | 0.54 | 0.44 | 0.03 | 14.61 | 0.03 |
| 76 | T-XJR | N：23º18.964' E：112º43.546' | 07/29/14 | 3.3 | 3.00 | 4.18 | 5.42 | 0.04 | 2.67 | 1.94 | 0.43 | 0.03 | 6.96 | 0.12 |
| 77 | T-XJR | N：23º17.430' E：112º45.794' | 07/29/14 | 7.1 | 3.06 | 2.39 | 49.69 | 0.05 | 0.78 | 1.22 | 0.48 | 0.05 | 8.69 | 0.09 |
| 78 | T-XJR | N：23º26.364' E：112º52.835' | 07/29/14 | 3.4 | 3.18 | 5.74 | 0.87 | 0.06 | 1.59 | 1.09 | 0.69 | 0.40 | 10.61 | 0.03 |
| 79 | T-XJR | N：23º11.753' E：112º48.475' | 07/29/14 | 2.5 | 2.85 | 3.89 | 0.38 | 0.06 | 1.75 | 1.54 | 0.74 | 0.18 | 9.77 | 0.03 |
| 80 | T-XJR | N：23º09.354' E：112º50.223' | 07/29/14 | 2.4 | 3.04 | 4.61 | 0.16 | 0.07 | 1.66 | 3.01 | 0.67 | 0.07 | 12.48 | 0.03 |
| 81 | XJR | N：23º06.474' E：112º47.941' | 07/29/14 | 2.9 | 3.42 | 6.88 | 0.15 | 0.08 | 1.68 | 0.63 | 0.48 | 0.05 | 15.98 | 0.03 |
| Low flow season | | | | | | | | | | | | | | |
| 1 | NPR | N：25º54.376' E：103º57.101' | 01/16/15 | 1.9 | 1.52 | 4.73 | 0.43 | 0.05 | 0.88 | 0.56 | 0.11 | 0.05 | 7.99 | 0.10 |
| 2 | NPR | N：25º36.971' E：103º49.532' | 01/16/15 | 4.8 | 2.25 | 10.58 | 0.68 | 0.25 | 3.04 | 0.75 | 0.37 | 0.07 | 30.16 | 0.09 |
| 3 | NPR | N：25º17.512' E：103º51.405' | 01/16/15 | 4.8 | 1.80 | 8.46 | 0.18 | 0.16 | 4.95 | 1.25 | 0.26 | 0.03 | 13.94 | 0.05 |
| 4 | NPR | N：25º00.589' E：103º38.313' | 01/16/15 | 4.1 | 2.23 | 9.19 | 0.17 | 0.22 | 2.93 | 1.59 | 0.63 | 0.05 | 15.68 | 0.05 |
| 5 | NPR | N：24º56.169' E：103º10.674' | 01/17/15 | 5.4 | 2.42 | 8.32 | 1.00 | 0.24 | 5.44 | 2.50 | 0.93 | 0.05 | 27.14 | 0.06 |
| 6 | NPR | N：24º13.314' E：103º06.415' | 01/17/15 | 4.6 | 2.32 | 8.89 | 0.38 | 0.18 | 3.53 | 1.22 | 0.90 | 0.04 | 31.41 | 0.10 |
| 7 | T-NPR | N：24º12.664' E：103º06.806' | 01/17/15 | 5.2 | 2.36 | 12.00 | 1.20 | 0.16 | 2.65 | 0.33 | 0.60 | 0.03 | 46.79 | 0.07 |
| 8 | T-NPR | N：24º13.778' E：103º23.804' | 01/17/15 | 4.2 | 2.53 | 9.33 | 0.25 | 0.15 | 3.33 | 0.93 | 0.38 | 0.04 | 15.65 | 0.05 |
| 9 | T-NPR | N：24º39.257' E：104º28.790' | 01/18/15 | 3.5 | 2.13 | 8.96 | 0.30 | 0.11 | 1.90 | 0.40 | 0.75 | 0.05 | 14.96 | 0.05 |
| 10 | NPR | N：24º39.293' E：104º28.732' | 01/18/15 | 3.8 | 2.20 | 9.06 | 0.43 | 0.13 | 2.41 | 2.62 | 0.77 | 0.04 | 14.90 | 0.06 |
| 11 | T-NPR | N：24º46.524' E：104º32.253' | 01/18/15 | 3.9 | 2.20 | 8.93 | 0.48 | 0.13 | 2.56 | 0.50 | 0.76 | 0.04 | 14.53 | 0.07 |
| 12 | T-NPR | N：25º11.607' E：104º54.663' | 01/19/15 | 3.9 | 2.06 | 7.74 | 0.37 | 0.10 | 3.39 | 2.38 | 0.69 | 0.04 | 12.29 | 0.07 |
| 13 | NPR | N：24º52.129' E：105º01.755' | 01/18/15 | 3.7 | 1.67 | 7.61 | 1.22 | 0.12 | 2.30 | 0.54 | 1.81 | 0.05 | 11.70 | 0.12 |
| 14 | T-BPR | N：26º00.141' E：104º30.067' | 01/15/15 | 4.4 | 2.38 | 9.02 | 0.32 | 0.11 | 2.60 | 4.48 | 2.44 | 0.07 | 28.73 | 0.09 |
| 15 | T-BPR | N：26º17.388' E：104º42.789' | 01/15/15 | 5.1 | 2.32 | 7.41 | 0.38 | 0.10 | 3.40 | 1.96 | 1.32 | 0.47 | 27.97 | 0.07 |
| 16 | T-BPR | N：26º22.931' E：105º00.733' | 01/14/15 | 19.1 | 1.58 | 5.21 | 134.97 | 1.57 | 3.46 | 1.15 | 0.83 | 0.06 | 14.53 | 0.05 |
| 17 | T-BPR | N：26º26.503' E：105º04.567' | 01/14/15 | 3.2 | 2.27 | 7.25 | 0.39 | 0.10 | 1.90 | 0.50 | 0.78 | 0.03 | 20.53 | 0.05 |
| 18 | T-BPR | N：26º10.138' E：105º11.266' | 01/14/15 | 4.2 | 2.00 | 6.23 | 0.22 | 0.11 | 4.57 | 0.81 | 0.69 | 0.08 | 16.23 | 0.06 |
| 19 | T-BPR | N：25º40.338' E：105º39.591' | 01/14/15 | 3.1 | 2.02 | 5.66 | 0.15 | 0.12 | 2.46 | 0.90 | 0.76 | 0.08 | 16.53 | 0.06 |
| 20 | T-BPR | N：26º00.774' E：105º40.083' | 01/14/15 | 5.1 | 1.57 | 8.67 | 0.39 | 0.17 | 4.85 | 0.94 | 0.77 | 0.04 | 31.10 | 0.07 |
| 21 | T-BPR | N：25º53.339' E：105º38.595' | 01/14/15 | 4.2 | 1.66 | 7.19 | 0.28 | 0.26 | 4.07 | 2.39 | 0.84 | 0.03 | 12.07 | 0.16 |
| 22 | T-BPR | N：25º52.383' E：105º40.367' | 01/14/15 | 3.5 | 1.58 | 8.37 | 0.17 | 0.16 | 2.23 | 0.43 | 0.58 | 0.03 | 16.25 | 0.05 |
| 23 | T-BPR | N：25º31.932' E：105º45.989' | 01/14/15 | 3.1 | 1.74 | 6.85 | 0.21 | 0.11 | 2.07 | 1.12 | 0.70 | 0.04 | 14.08 | 0.09 |
| 24 | T-BPR | N：25º28.012' E：105º46.908' | 01/13/15 | 3.1 | 1.91 | 6.69 | 0.29 | 0.12 | 2.19 | 0.59 | 0.77 | 0.04 | 15.18 | 0.07 |
| 25 | T-BPR | N：25º23.054' E：105º45.477' | 01/13/15 | 3.3 | 1.85 | 7.98 | 0.23 | 0.16 | 1.87 | 0.53 | 0.87 | 0.03 | 17.24 | 0.07 |
| 26 | T-BPR | N：25º22.678' E：105º47.127' | 01/13/15 | 3.6 | 1.82 | 5.99 | 0.21 | 0.12 | 3.41 | 5.59 | 0.70 | 0.06 | 14.41 | 0.07 |
| 27 | T-BPR | N：25º06.965' E：105º51.618' | 01/13/15 | 3.0 | 1.51 | 7.14 | 0.21 | 0.21 | 1.76 | 0.55 | 0.84 | 0.07 | 10.68 | 0.06 |
| 28 | T-BPR | N：25º07.095' E：105º51.757' | 01/13/15 | 3.2 | 1.80 | 6.48 | 0.46 | 0.12 | 2.20 | 0.43 | 0.78 | 0.06 | 15.20 | 0.08 |
| 29 | T-BPR | N：25º04.399' E：105º57.121' | 01/13/15 | 13.5 | 1.62 | 6.29 | 0.51 | 0.12 | 22.70 | 43.88 | 0.69 | 0.05 | 13.74 | 0.14 |
| 30 | T-BPR | N：25º02.840' E：105º55.993' | 01/13/15 | 3.5 | 1.65 | 6.20 | 0.19 | 0.11 | 3.05 | 0.90 | 0.66 | 0.05 | 15.54 | 0.07 |
| 31 | T-BPR | N：24º09.598' E：106º05.519' | 01/12/15 | 3.3 | 1.60 | 7.22 | 0.24 | 0.13 | 2.22 | 0.65 | 0.56 | 0.06 | 18.39 | 0.06 |
| 32 | T-BPR | N：24º56.984' E：106º08.133' | 01/12/15 | 3.0 | 1.68 | 6.49 | 0.30 | 0.11 | 2.01 | 0.55 | 0.77 | 0.07 | 13.93 | 0.06 |
| 33 | NPR | N：24º56.894' E：106º08.749' | 01/12/15 | 3.0 | 1.77 | 6.13 | 0.39 | 0.12 | 1.97 | 0.68 | 0.93 | 0.09 | 14.68 | 0.07 |
| 34 | HSR | N：24º59.741' E：106º09.535' | 01/12/15 | 2.9 | 1.64 | 6.82 | 0.31 | 0.10 | 1.54 | 0.48 | 0.75 | 0.05 | 14.68 | 0.06 |
| 35 | T-HSR | N：24º58.122' E：106º09.510' | 01/12/15 | 2.9 | 1.73 | 6.83 | 0.25 | 0.10 | 1.66 | 0.45 | 0.76 | 0.06 | 14.72 | 0.06 |
| 36 | HSR | N：25º09.847' E：106º37.839' | 01/22/15 | 4.6 | 2.32 | 7.52 | 0.19 | 0.13 | 4.61 | 1.54 | 0.73 | 0.07 | 13.83 | 0.09 |
| 37 | T-HSR | N：26º05.865' E：106º38.245' | 01/19/15 | 4.0 | 1.64 | 8.03 | 2.14 | 0.14 | 3.15 | 0.55 | 0.77 | 0.05 | 8.87 | 0.07 |
| 38 | T-HSR | N：25º27.515' E：106º46.701' | 01/22/15 | 3.9 | 1.90 | 8.11 | 1.81 | 0.12 | 2.92 | 1.65 | 0.68 | 0.04 | 11.74 | 0.05 |
| 39 | T-HSR | N：25º04.082' E：106º57.780' | 01/22/15 | 3.8 | 2.00 | 7.16 | 0.68 | 0.11 | 3.33 | 1.46 | 0.48 | 0.04 | 13.16 | 0.07 |
| 40 | HSR | N：24º59.919' E：107º10.015' | 01/22/15 | 2.8 | 2.19 | 6.47 | 0.19 | 0.12 | 1.94 | 0.40 | 0.66 | 0.03 | 12.00 | 0.05 |
| 41 | T-HSR | N：24º50.647' E：107º19.850' | 01/23/15 | 3.2 | 2.07 | 7.55 | 0.20 | 0.11 | 1.99 | 0.36 | 0.56 | 0.03 | 14.63 | 0.07 |
| 42 | T-HSR | N：24º31.637' E：107º45.463' | 01/23/15 | 3.7 | 1.98 | 7.14 | 0.18 | 0.10 | 3.49 | 3.93 | 0.37 | 0.04 | 8.39 | 0.05 |
| 43 | T-HSR | N：24º34.169' E：107º49.052' | 01/23/15 | 4.3 | 1.81 | 7.15 | 1.49 | 0.14 | 3.31 | 2.71 | 0.71 | 0.25 | 9.67 | 0.05 |
| 44 | T-QJR | N：24º42.316' E：108º02.125' | 01/23/15 | 11.2 | 2.09 | 8.49 | 0.26 | 0.10 | 17.91 | 21.81 | 0.44 | 0.03 | 9.03 | 0.04 |
| 45 | T-QJR | N：24º34.145' E：108º28.336' | 01/23/15 | 4.1 | 2.11 | 9.09 | 2.80 | 0.47 | 2.60 | 0.58 | 0.40 | 0.06 | 10.17 | 0.08 |
| 46 | T-QJR | N：24º30.130' E：108º37.738' | 01/23/15 | 3.5 | 2.13 | 8.03 | 0.86 | 0.11 | 2.28 | 0.40 | 0.50 | 0.05 | 13.91 | 0.06 |
| 47 | T-QJR | N：24º32.325' E：109º14.632' | 01/24/15 | 3.1 | 1.94 | 7.73 | 0.16 | 0.10 | 1.95 | 0.37 | 0.35 | 0.05 | 11.15 | 0.04 |
| 48 | T-QJR | N：24º32.698' E：109º15.904' | 01/24/15 | 1.9 | 2.06 | 3.34 | 3.96 | 0.05 | 0.80 | 0.44 | 0.24 | 0.03 | 11.22 | 0.04 |
| 49 | T-QJR | N：24º32.082' E：109º14.962' | 01/24/15 | 2.3 | 1.92 | 5.48 | 0.20 | 0.07 | 1.46 | 0.38 | 0.30 | 0.03 | 9.29 | 0.04 |
| 50 | T-QJR | N：23º50.551' E：109º31.877' | 01/24/15 | 2.4 | 2.12 | 4.60 | 0.42 | 0.06 | 1.24 | 0.53 | 0.35 | 0.04 | 29.92 | 0.05 |
| 51 | QJR | N：23º40.025' E：109º40.139' | 01/24/15 | 2.6 | 2.02 | 6.04 | 0.25 | 0.09 | 1.72 | 0.56 | 0.41 | 0.04 | 10.27 | 0.06 |
| 52 | QJR | N：23º36.249' E：109º38.862' | 01/24/15 | 3.0 | 2.17 | 6.86 | 0.19 | 0.10 | 1.99 | 1.60 | 0.51 | 0.03 | 11.75 | 0.05 |
| 53 | QJR | N：23º24.696' E：110º04.310' | 01/24/15 | 12.8 | 2.06 | 6.17 | 0.81 | 0.10 | 22.71 | 0.58 | 0.45 | 0.04 | 10.66 | 0.03 |
| 54 | T-XUR | N：22º49.966' E：108º05.609' | 01/27/15 | 3.6 | 2.24 | 9.00 | 1.06 | 0.09 | 1.75 | 7.28 | 0.24 | 0.02 | 18.50 | 0.06 |
| 55 | T-XUR | N：22º50.682' E：108º05.743' | 01/27/15 | 3.5 | 2.65 | 8.12 | 0.48 | 0.13 | 1.97 | 0.66 | 0.54 | 0.05 | 22.27 | 0.06 |
| 56 | T-XUR | N：22º49.395' E：108º11.372' | 01/27/15 | 3.2 | 2.55 | 7.72 | 0.69 | 0.11 | 1.83 | 0.55 | 0.40 | 0.05 | 11.34 | 0.06 |
| 57 | T-XUR | N：22º54.480' E：109º34.174' | 01/27/15 | 3.7 | 3.00 | 8.32 | 0.55 | 0.12 | 1.87 | 0.50 | 0.91 | 0.16 | 19.03 | 0.05 |
| 58 | T-XUR | N：23º22.370' E：110º04.851' | 01/24/15 | 3.1 | 2.61 | 7.26 | 0.75 | 0.10 | 1.84 | 3.07 | 0.52 | 0.03 | 15.54 | 0.04 |
| 59 | XUR | N：23º26.103' E：110º07.647' | 01/25/15 | 3.2 | 2.31 | 7.91 | 0.45 | 0.21 | 1.77 | 0.51 | 0.55 | 0.04 | 12.93 | 0.04 |
| 60 | XUR | N：23º25.594' E：110º31.940' | 01/25/15 | 3.2 | 2.32 | 7.10 | 0.82 | 0.11 | 2.06 | 0.49 | 0.55 | 0.04 | 17.25 | 0.04 |
| 61 | T-XUR | N：23º24.980' E：110º31.817' | 01/25/15 | 43.9 | 2.18 | 4.32 | 209.01 | 0.24 | 37.35 | 1.10 | 0.17 | 0.06 | 32.34 | 0.11 |
| 62 | XUR | N：23º25.359' E：110º32.275' | 01/25/15 | 3.7 | 2.26 | 5.97 | 0.25 | 0.09 | 3.51 | 16.99 | 0.48 | 0.05 | 12.52 | 0.04 |
| 63 | XUR | N：23º21.571' E：110º51.468' | 01/25/15 | 2.9 | 2.40 | 6.76 | 0.24 | 0.11 | 1.75 | 0.56 | 0.52 | 0.04 | 15.88 | 0.04 |
| 64 | T-XUR | N：23º20.937' E：110º53.646' | 01/25/15 | 20.7 | 2.11 | 4.05 | 9.49 | 0.17 | 34.86 | 115.73 | 0.21 | 0.03 | 20.86 | 0.04 |
| 65 | XUR | N：23º23.557' E：110º55.022' | 01/25/15 | 2.4 | 2.03 | 4.88 | 1.60 | 0.12 | 1.30 | 0.49 | 0.37 | 0.03 | 16.46 | 0.05 |
| 66 | T-XJR | N：23º30.364' E：110º19.704' | 01/25/15 | 2.9 | 2.26 | 5.22 | 0.51 | 0.08 | 2.39 | 1.43 | 0.41 | 0.04 | 18.59 | 0.06 |
| 67 | XJR | N：23º28.637' E：110º20.612' | 01/25/15 | 5.4 | 2.22 | 5.73 | 1.21 | 0.10 | 7.08 | 1.64 | 0.52 | 0.04 | 18.57 | 0.05 |
| 68 | T-XJR | N：23º27.087' E：111º30.237' | 01/25/15 | 12.8 | 2.19 | 4.03 | 0.88 | 0.05 | 1.80 | 6.15 | 95.75 | 0.31 | 15.01 | 0.05 |
| 69 | XJR | N：23º20.329' E：111º31.631' | 01/26/15 | 3.1 | 2.22 | 6.85 | 0.23 | 0.11 | 1.84 | 1.39 | 1.46 | 0.04 | 20.09 | 0.05 |
| 70 | T-XJR | N：23º07.208' E：111º49.460' | 01/26/15 | 3.3 | 2.35 | 4.61 | 0.62 | 0.05 | 2.08 | 12.84 | 5.25 | 0.05 | 17.06 | 0.04 |
| 71 | T-XJR | N：23º09.157' E：111º51.424' | 01/26/15 | 3.1 | 2.33 | 2.68 | 15.20 | 0.06 | 0.47 | 0.60 | 0.38 | 0.03 | 17.14 | 0.12 |
| 72 | XJR | N：23º07.908' E：111º55.040' | 01/26/15 | 3.8 | 2.48 | 6.42 | 0.89 | 0.11 | 2.74 | 3.73 | 0.96 | 0.05 | 17.24 | 0.27 |
| 73 | T-XJR | N：23º05.709' E：112º07.908' | 01/26/15 | 1.5 | 2.36 | 2.51 | 0.71 | 0.03 | 0.93 | 0.41 | 0.30 | 0.02 | 13.76 | 0.04 |
| 74 | T-XJR | N：23º11.024' E：112º19.212' | 01/26/15 | 33.5 | 2.37 | 3.62 | 267.33 | 0.64 | 1.38 | 3.20 | 0.30 | 0.04 | 10.24 | 0.60 |
| 75 | XJR | N：23º02.455' E：112º25.236' | 01/26/15 | 2.8 | 2.49 | 6.10 | 0.74 | 0.09 | 1.66 | 0.79 | 0.55 | 0.04 | 16.61 | 0.03 |
| 76 | T-XJR | N：23º18.964' E：112º43.546' | 01/27/15 | 3.2 | 2.48 | 5.28 | 2.90 | 0.06 | 2.75 | 1.01 | 0.48 | 0.02 | 12.31 | 0.04 |
| 77 | T-XJR | N：23º17.430' E：112º45.794' | 01/27/15 | 2.8 | 2.50 | 3.84 | 0.53 | 0.04 | 2.93 | 4.84 | 0.53 | 0.04 | 13.20 | 0.03 |
| 78 | T-XJR | N：23º26.364' E：112º52.835' | 01/27/15 | 7.6 | 2.46 | 4.36 | 1.01 | 0.10 | 3.00 | 2.23 | 1.07 | 2.09 | 14.14 | 0.05 |
| 79 | T-XJR | N：23º11.753' E：112º48.475' | 01/26/15 | 4.9 | 2.47 | 4.62 | 1.06 | 0.09 | 5.23 | 1.87 | 1.27 | 0.15 | 42.27 | 0.03 |
| 80 | T-XJR | N：23º09.354' E：112º50.223' | 01/26/15 | 3.4 | 2.33 | 4.47 | 0.45 | 0.09 | 3.51 | 1.78 | 0.94 | 0.10 | 13.45 | 0.04 |
| 81 | XJR | N：23º06.474' E：112º47.941' | 01/26/15 | 3.8 | 2.46 | 6.32 | 1.32 | 0.09 | 3.49 | 1.08 | 0.60 | 0.04 | 16.09 | 0.05 |
| NPR, Nanpanjiang River; HSR, Hongshuihe River; QJR, Qianjiang River; XUJ, Xunjiang River; XJR, Xijiang River. | | | | | | | | | | | | | | |
| T-NPR, T-BPR, T-HSR, T-QJR, T-XUJ and T-XJR represents the tributaries of Nanpanjiang River, Beipanjiang River, Hongshuihe River, Qianjiang River, Xunjiang River and Xijiang River. | | | | | | | | | | | | | | |
