# Supplementary material for: Geochemical characteristics of dissolved heavy metals in Zhujiang River, Southwest China: spatial-temporal distribution, source, export flux estimation, and a water quality assessment
Source: PeerJ. 2019 Mar 13;7:e6578. doi: 10.7717/peerj.6578 (PMC6420802; doi:10.7717/peerj.6578)
Supplement: Supplemental Information 2 — All the standard values are from Chinese surface water standards (GB 3838-2002). [file peerj-07-6578-s002.docx]

| Grade^a^ | Cr | Cu | Cd | Pb | pH | DO |
| --- | --- | --- | --- | --- | --- | --- |
| Ⅰ | 10 | 10 | 1 | 10 | 6.0-9.0 | 7.5 |
| Ⅱ | 50 | 1000 | 5 | 10 | 6.0-9.0 | 6.0 |
| Ⅲ | 50 | 1000 | 5 | 50 | 6.0-9.0 | 5.0 |
| Ⅳ | 50 | 1000 | 5 | 50 | 6.0-9.0 | 3.0 |
| Ⅴ | 100 | 1000 | 10 | 100 | 6.0-9.0 | 2.0 |
| ^a^ Chinese surface water standards (GB 3838-2002). | | | | | | |
