# Supplementary material for: Geochemical characteristics of dissolved heavy metals in Zhujiang River, Southwest China: spatial-temporal distribution, source, export flux estimation, and a water quality assessment
Source: PeerJ. 2019 Mar 13;7:e6578. doi: 10.7717/peerj.6578 (PMC6420802; doi:10.7717/peerj.6578)
Supplement: Supplemental Information 3 — The dissolved heavy metal concentrations of the Yellow River and the mouth of Mississippi River are expressed as a range of maximum and minimum values. [file peerj-07-6578-s003.docx]

| River | V | Cr | Mn | Co | Ni | Cu | Mo | Cd | Ba | Pb |
| --- | --- | --- | --- | --- | --- | --- | --- | --- | --- | --- |
| Low flow season (This study) | 2.13 | 6.85 | 0.45 | 0.11 | 2.39 | 0.90 | 0.63 | 0.04 | 14.72 | 0.06 |
| High flow season (This study) | 2.59 | 7.52 | 0.40 | 0.11 | 2.08 | 0.77 | 0.44 | 0.03 | 12.50 | 0.04 |
| Yangtze River, China ^a^ |  |  | 1.00 |  | 0.15 | 1.66 |  | 0.003 |  | 0.05 |
| Yellow River, China^a^ |  |  | 0.55-2.2 | 0.01-0.03 | 0.30-0.59 | 0.96-1.60 |  | 0.001-0.006 |  | 0.01-4.10 |
| Seine River, France^a^ | 2.85 | 11.46 | 3.76 | 0.18 | 5.06 | 3.53 |  | 0.06 | 32.00 | 0.22 |
| Harz Mountains River, Germany^a^ | 0.4 | <0.85 | 48 | 0.26 | 0.92 | 0.82 |  | 0.42 | 13.00 | 3.80 |
| Ottawa River, Canada^a^ | 0.341 |  | 14.86 | 0.0746 | 0.83 | 1.144 | 0.199 | 0.0207 | 15.00 | 0.11 |
| Mouth of Mississippi River, America^a^ | 0.82-1.84 |  | 0.66-1.82 |  | 1.12-1.77 | 1.60-2.24 | 1.63-2.69 |  | 62.00 | 0.011-0.016 |
| Amazon River, South America^a^ | 0.70 | 0.72 | 50.73 | 0.18 | 0.74 | 1.46 | 0.18 | 0.18 | 21.00 | 0.06 |
| World River average^a^ | 0.71 | 0.70 | 34.00 | 0.15 | 0.80 | 1.48 | 0.42 | 0.08 | 23.00 | 0.08 |
| ^a^ Gaillardet et al., 2014 | | | | | | | | | | |
